# Impacts of seasonality on gene expression in the Chinese horseshoe bat

**DOI:** 10.1002/ece3.8923

**Published:** 2022-05-13

**Authors:** Wenli Chen, Xiuguang Mao

**Affiliations:** ^1^ 12655 School of Ecological and Environmental Sciences East China Normal University Shanghai China

**Keywords:** bats, circannual, immunity, seasonal changes, transcriptome

## Abstract

Seasonality can cause changes in many environmental factors which potentially affects gene expression. Here, we used a bat species (*Rhinolophus sinicus*) from eastern China as a model to explore the molecular mechanisms of seasonal effects, in particular during phenological shifts in the spring and autumn. Based on the analysis of 45 RNA‐seq samples, we found strong seasonal effects on gene expression, with a large number of genes identified as either specific or biased to each season. Weighted gene co‐expression network analysis also identified multiple modules significantly associated with each season. These seasonal genes were further enriched into different functional categories. Consistent with effects of phenological shifts on bats, we found that genes related to promoting food intake were highly expressed in both autumn and spring. In addition, immunity genes were also highly expressed in both seasons although this seasonal immune response had tissue specificity in different seasons. In female bats, genes related to the delay of ovulation (e.g., *NPPC*, natriuretic peptide precursor type C) were highly expressed in October, while genes associated with the promotion of reproduction (e.g., *DIO2*, iodothyronine deiodinase 2) were biasedly expressed in April. Lastly, we found multiple known core clock genes in both October‐biased and April‐biased expressed genes, which may be involved in regulating the start and end of hibernation, respectively. Overall, together with studies in other land and aquatic animals, our work supports that seasonal gene expression variations may be a general evolutionary response to environmental changes in wild animals.

## Graphical abstract

We found strong seasonal effects on gene expression in multiple tissues in the Chinese horseshoe bat. Consistent with previous studies, our work revealed that molecular changes of the immune function were coincided with phenological shifts in the spring and autumn and also supported an important role of hormone genes in regulating organism's seasonal physiology. Overall, our work supports that seasonal gene expression variations may be a general evolutionary response to environmental changes in wild animals.

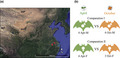

## 1 INTRODUCTION

Seasonality, seasonal cycle through a year, can cause changes in many environmental factors, such as differences in day‐length, humidity, temperature, and food availability (Lisovski et al., 2017). These factors can promote the evolution of morphological and physiological adaptations (Williams et al., 2017), which can be partially achieved by regulation of gene expression (Cheviron & Swanson, 2017; Daniels et al., 2014; Harrison et al., 2012). Numerous studies in humans have discovered that several aspects of human physiology and diseases coincide with seasonal changes in gene expression (De Jong et al., 2014; Dopico et al., 2015; Goldinger et al., 2015; Wucher et al., 2021). In wild animals, however, most previous studies on seasonality influence on gene expression focused on insects with short generation times, such as Drosophila (Zhao et al., 2016), butterflies (Oostra et al., 2018), and bees (Bresnahan et al., 2022). There have been few studies on animals with long generation time (but see Cheviron & Swanson, 2017; Schwartz et al., 2013; Sharma et al., 2020; Van Dolah et al., 2015). Lack of knowledge in these groups hinders our understanding of the general principles underlying seasonal adaptations across animal species (see also Williams et al., 2017).

Bats (Chiroptera), the second largest mammalian order, comprises more than 1400 species (Simmons & Cirranello, 2020) and have wide distributions across the world. Seasonality also has a large effect on bats’ activity patterns and survival (Heideman & Utzurrum, 2003; Sendor & Simon, 2003). In particular, bats inhabiting temperate regions usually hibernate in winter in response to low temperature and limited food availability (Nowack et al., 2017; Webb et al., 1996). In addition, similar to most insects (Bresnahan et al., 2022; Zhao et al., 2016), hibernating bats also delay reproduction till spring (Oxberry, 1979). Previous studies on gene expression changes in bats mainly focused on comparisons of samples in winter torpid state and summer active state (Lei et al., 2014; Sun et al., 2020; Xiao et al., 2015). However, phenological shifts in the spring and autumn, such as the day length and temperature, can cause large impacts on fitness compared with other seasons (Levy et al., 2016; Williams et al., 2017). To fully understand the molecular mechanism of seasonal effects, samples collected in spring and autumn should be studied (see Schwartz et al., 2013).

In this study, we used a bat species (*Rhinolophus sinicus*) from eastern China, which can hibernate in winter, as a model to explore the molecular mechanisms of seasonal effects in wild land mammals. We sampled *R*. *sinicus* individuals in April and October, representing spring and autumn, respectively. April coincides with the end of hibernation in bats, during which both males and females feed a lot at night. In addition, females begin to be pregnant and prepare for reproduction. Similar to April, both sexes of bats also feed on a lot in October to store fat for the start of hibernation. In addition, oocytes in females were found to maintain meiotic arrest in autumn (Chanda et al., 2003; Wang et al., 2008). Lastly, similar to other hibernating mammals (e.g., thirteen‐lined ground squirrel), in bats, timing of the start and the end of hibernation may also depend on circannual clock (Helm et al., 2013; Schwartz et al., 2013).

By performing differential expression analysis and weighted gene co‐expression network analysis (WGCNA), the main aim of this study was to identify genes and pathways associated with seasonal changes. We collected eight and seven individuals in April and October, respectively. To test for tissue specificity of gene expression, we sampled three different tissues (brain, cochlea, and liver) for each individual. Brain and liver were commonly used to investigate seasonal differences in gene expression (e.g., Gillen et al., 2021; Johnston et al., 2016; Schwartz et al., 2013; Xiao et al., 2015; Zhao et al., 2016). In this study, we also included cochlea tissue because previous studies have indicated seasonal changes in echolocation calls and auditory processing in bats (Grilliot et al., 2014; Miller et al., 2016). Based on the similarity and difference in phenological effects between spring and autumn (see above), we further tested for the following two predictions. First, genes and pathways involved in promoting food intake and rhythmic process will be found to be highly expressed in both April and October. Second, in females, some genes with April‐biased expression may be related to the stimulation of reproduction, while some with October‐biased expression may be associated with the delay of ovulation. Overall, this study aimed to provide insights into the mechanisms underlying adaptation to seasonal variation, in particular during rapid global climatic and environmental changes.

## 2 MATERIAL AND METHODS

### 2.1 Sampling and mRNA‐seq data collection

**FIGURE 1:**
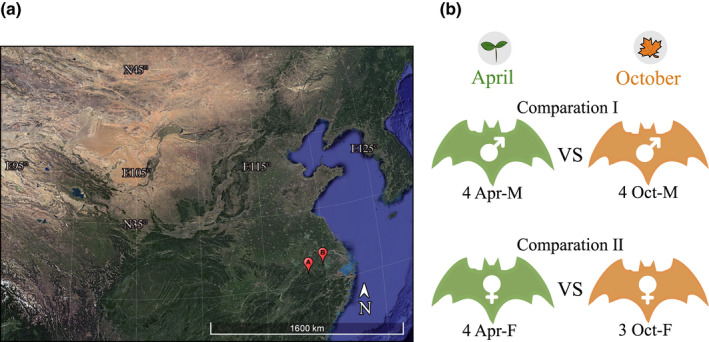
Sampling and experimental design. (a) Sampling locality of *Rhinolophus sinicus* in this study (A: Anhui; B: Jiangsu, see Table 1). (b) Experimental design. Samples were collected in two seasons (April and October) and were divided into four groups: April males (Apr‐M), April females (Apr‐F), October males (Oct‐M), and October females (Oct‐F)

To investigate seasonal variation of transcriptional profiles, we sampled bats of *Rhinolophus sinicus* in eastern China (Figure 1a) in April 2018 and October 2020, representing spring and autumn months, respectively. Specifically, bats were captured using mist nets at the cave entrance when they flew out for food at dusk. We then checked for the joint of the fifth finger which remains swollen in juveniles and becomes knobbly in adults. Only adult bats were collected. Finally, we chose four bats of each sex in April and four males and three females in October as representative individuals (Figure 1b; Table 1). All bats were euthanized by cervical dislocation. For each bat, we collected tissues of brain, cochleae, and liver. All tissues were frozen immediately in liquid nitrogen and stored in a −80°C freezer. Our sampling and tissue collection procedures were approved by the National Animal Research Authority, East China Normal University (approval ID Rh20200801).

**TABLE 1:** Detailed information of samples used in this study. Apr‐F, Apr‐M, Oct‐F, and Oct‐M represent April females, April males, October females, and October males, respectively

Sample ID	Sex	Sampling locality	Sampling date	Group
180401	Male	Jiangsu, China	April 19, 2018	Apr‐M
180404	Male	Jiangsu, China	April 19, 2018	Apr‐M
180406	Male	Jiangsu, China	April 19, 2018	Apr‐M
180411	Male	Jiangsu, China	April 19, 2018	Apr‐M
180402	Female	Jiangsu, China	April 19, 2018	Apr‐F
180403	Female	Jiangsu, China	April 19, 2018	Apr‐F
180409	Female	Jiangsu, China	April 19, 2018	Apr‐F
180410	Female	Jiangsu, China	April 19, 2018	Apr‐F
20201024	Male	Jiangsu, China	October 17, 2020	Oct‐M
20201037	Male	Anhui, China	October 24, 2020	Oct‐M
20201039	Male	Anhui, China	October 24, 2020	Oct‐M
20201040	Male	Anhui, China	October 24, 2020	Oct‐M
20201029	Female	Jiangsu, China	October 17, 2020	Oct‐F
20201033	Female	Jiangsu, China	October 17, 2020	Oct‐F
20201038	Female	Anhui, China	October 24, 2020	Oct‐F

For each tissue sample, total RNA was extracted using TRIzol (Life Technologies Corp). The RNA integrity number (RIN) and concentration were determined using the Agilent 2100 Bioanalyzer system. A total of 45 sequencing libraries (15 individuals × 3 tissues) were created with NEBNext^®^ UltraTM RNA Library Prep Kit for Illumina^®^ (NEB, USA) and sequenced on an Illumina HiSeq X Ten platform (paired‐end 150 bp).

### 2.2 RNA‐Seq data trimming and mapping

Raw sequencing reads of each sample were processed using TRIMMOMATIC version 0.38 (Bolger et al., 2014) with the parameters of SLIDINGWINDOW:4:20. All reads were further trimmed to 120 bp. For each sample, filtered reads were mapped to a chromosome‐level genome of a male *R*. *sinicus* (unpublished data from J Dong) using HISAT2 version 2.2.0 (Kim et al., 2015) with default settings. Then sorted BAM files were created based on SAM files using SAMtools v1.11 (Li et al., 2009). For each sample, mapped reads were quantified using Featurecount Version 2.0.1 (Liao et al., 2014) with default settings.

### 2.3 Clustering analysis

To compare expression patterns across samples, raw counts of each sample were normalized using DESeq function in DESeq2 package first (Love et al., 2014). Then we filtered out those genes with low expression (average CPM < 1 in all samples), resulting in a total of 13,781 expressed genes.

To explore the overall expression pattern across all 45 samples, we first conducted principal component analysis (PCA) with PlotPCA function in DESeq2 package. Second, we performed hierarchical clustering and heatmaps using the R package pvclust v2.2‐0 (Suzuki & Shimodaira, 2006) and pheatmap v1.0.12 (Kolde, 2012), respectively. Hierarchical clustering was performed based on Euclidean distances of rlog‐transformed read counts. The reliability of each node was determined using bootstrap resampling (1000 replicates).

### 2.4 Differential expression analysis

To compare gene expression differences between seasons in each sex, we divided the 15 individuals into four groups (Figure 1b; Table 1): April males (Apr‐M), April females (Apr‐F), October males (Oct‐M), and October females (Oct‐F). A total of six comparisons were included. For each comparison, we first identified season‐specific genes, including Apr‐specific genes and Oct‐specific genes, by comparing the list of genes expressed in each of the two groups. Then, shared genes in both seasons of each sex were used to perform differential expression (DE) analysis to identify season‐biased genes, including Apr‐biased and Oct‐biased genes. DE analyses were conducted using the DESeq2 package (version: 1.30.1, Love et al., 2014). Season‐biased (Apr‐biased or Oct‐biased) genes were determined with the *p*‐value <.05 after Benjamini and Hochberg adjustment for multiple tests (padj < 0.05, Benjamini & Hochberg, 1995). To further explore the grouping of individuals sampled from different seasons, hierarchical clustering analysis was also performed based on season‐biased genes in each tissue using the same procedures as above.

### 2.5 Weighted gene co‐expression network analysis

To identify co‐expressed clusters of genes that respond to each season, we performed Weighted gene co‐expression network analysis (WGCNA) (the WGCNA package, Version 1.70.3; Langfelder & Horvath, 2008) on each tissue. Before the WGCNA, raw counts of all expressed genes across samples were normalized using DESeq2. Then WGCNA was performed step by step. First, we chose a soft threshold power for each tissue (brain: 7; cochlea: 7; liver: 10) to achieve values of 0.80 for a scale‐free topology fit index. Second, modules were identified and an adjacency matrix was calculated using the corresponding soft threshold power. To minimize effects of noise and spurious associations, we transformed the adjacency matrix into Topological Overlap Matrix (TOM) and calculated the corresponding dissimilarity by subtracting it from 1. Genes with similar co‐expression patterns across samples were grouped using hierarchical clustering of dissimilarity among the topological overlap measures. Co‐expressed modules were determined using a dynamic tree‐cutting algorithm setting with a minimum module size of 30. Third, highly similar modules were merged by a module dissimilarity threshold of 0.25. Finally, correlations between external qualitative traits (0 or 1), including sex (male: 1, female: 0) and season (April: 1, October: 0), and gene expression of each module were quantified by calculating Pearson correlations and *p*‐values. Significant module–trait relationships were determined with *p*‐value ≤.01. In each significant module, we only focus on hub genes with the absolute value of gene significance (GS) >0.2 and the absolute value of module membership (MM) >0.8.

### 2.6 Functional gene ontology analysis

To explore the biological functions of the season‐specific genes, season‐biased genes, and hub genes identified in the WGCNA, functional enrichment analysis was performed using Matecaspe (http://metascape.org) with the Custom Analysis module (Zhou et al., 2019). The enrichment background list included a total of 14,448 expressed genes in three tissues. Significantly enriched gene ontology (GO) terms and pathways were determined with q‐value <0.05 calculated by accounting for multiple tests in the Benjamini‐Hochberg procedure (Hochberg & Benjamini, 1990).

## 3 RESULTS

Using the high‐through sequencing technology, we generated an average of 23,525,856 read pairs 150 bp long per sample. After quality control, an average of 19,614,593 clean reads pairs were retained per sample with an overall mapping rate of 98% to the reference genome (Dryad file Table S1).

### 3.1 Samples tend to cluster with the sampling season in each tissue

**FIGURE 2:**
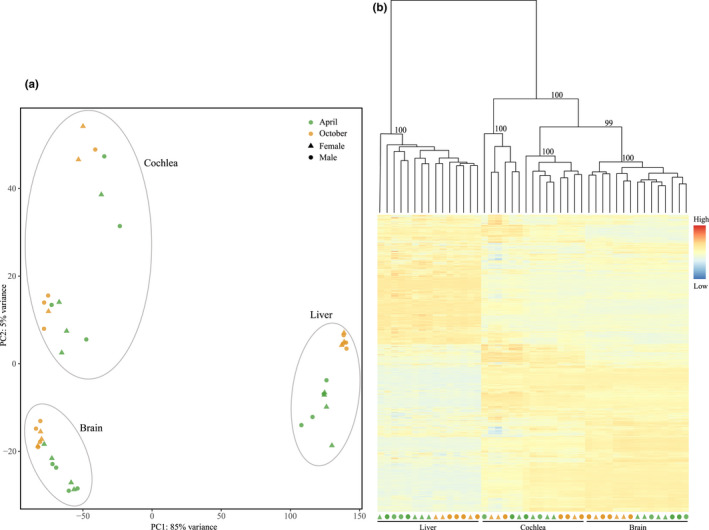
Clustering analysis for all 45 samples based on normalized gene expression matrix of 13,781 genes. (a) Principal component analysis; (b) hierarchical clustering and heatmap. Numbers on each node indicate the bootstrap support values

**FIGURE 3:**
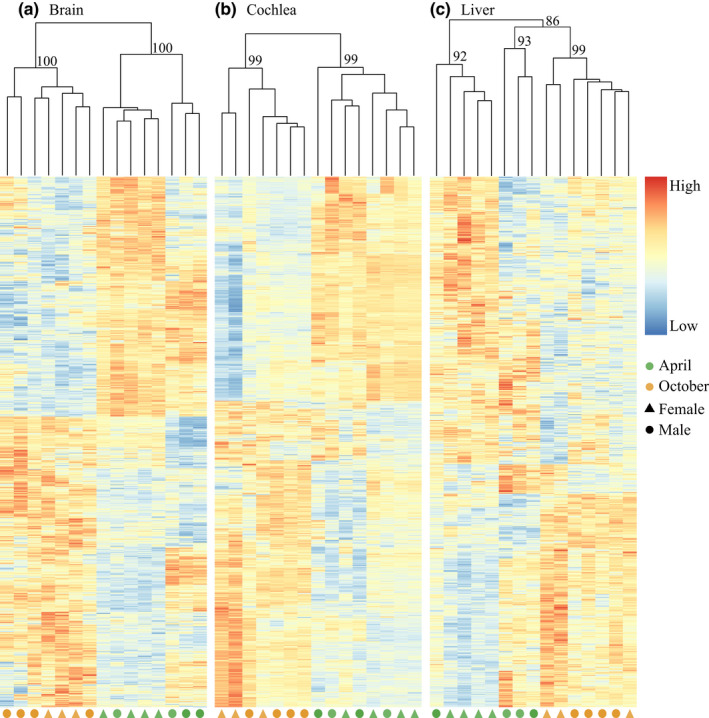
Hierarchical clustering and heatmap showing patterns of expression of season‐biased expressed genes in the brain (a), cochlea (b), and liver (c). Numbers on each node indicate the bootstrap support values

Principle components analysis (PCA) divided all samples into three clusters corresponding to each tissue (Figure 2a). Specifically, PC1 separated liver samples from cochlea and brain samples with 85% of gene expression variation, while PC2 separated cochlea and brain samples with 5% of gene expression variation. In liver and brain tissues, samples were further divided into two clusters based on each season, while in cochlea, April and October samples clustered. It was notable that the Cochlea samples were further separated by PC2 into two distinct clusters, which do not relate to either sex or season. Consistent with the PCA results, hierarchical clustering analysis also revealed that cochlea and brain samples clustered with bootstrap support value of 100 and were separated from liver samples (Figure 2b). Similarly, samples of each season were more likely classified together in the liver and brain, while they were mixed in the cochlea. This seasonal clustering pattern was more clearly shown in the hierarchical clustering analysis based on season‐biased genes in each tissue (Figure 3).

### 3.2 General patterns of seasonal genes identified in this study

In general, we identified more Apr‐specific genes than Oct‐specific genes in each tissue (Dryad file Figure S1a and Dryad file Table S2). However, for season‐biased genes, we found more Oct‐biased genes than Apr‐biased genes in all three tissues except for the liver (Dryad file Figure S1b and Dryad file Table S3). These patterns were observed in genes expressed in each individual sex. However, for all differentially expressed genes (DEGs=the total of season‐specific and season‐biased genes), we found similar numbers of DEGs in the brain and cochlea in each sex, whereas in the liver, about two‐fold numbers of DEGs were observed in April than in October (Dryad file Figure S1c).

In each tissue, males and females exhibited similar numbers of season‐specific (Apr‐ or Oct‐specific) genes, while females showed much higher number of season‐biased (Apr‐ or Oct‐biased) genes than males except for April‐biased genes in the cochlea (Dryad file Figure S1a,b).

### 3.3 Identification of genes and pathways associated with autumn

**FIGURE 4:**
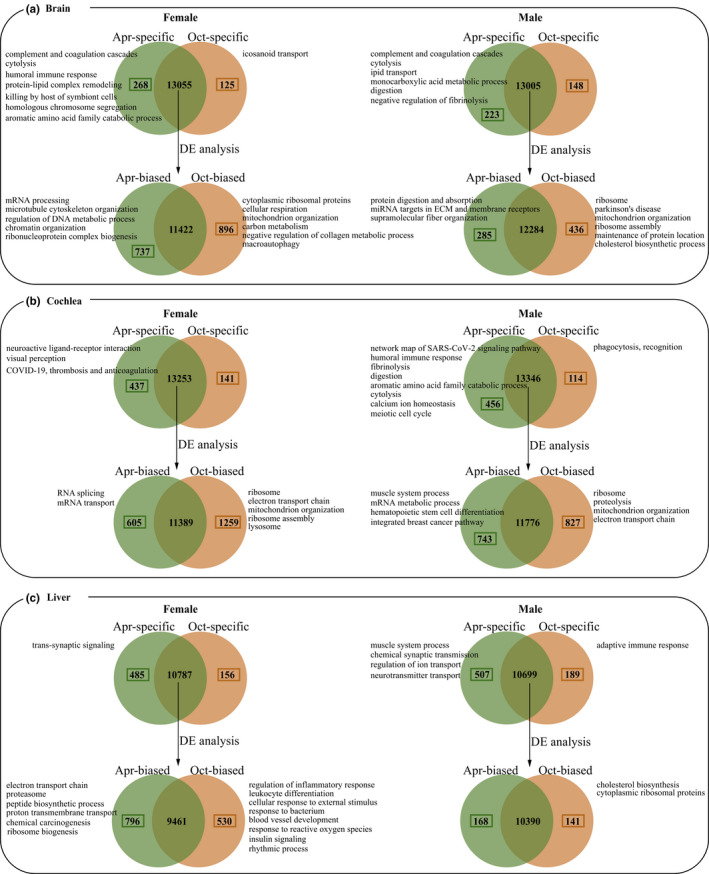
Identification and characterization of season‐specific and season‐biased expressed genes in the brain (a), cochlea (b),and liver (c). The representative GO terms or pathways identified on each category genes were shown

**FIGURE 5:**
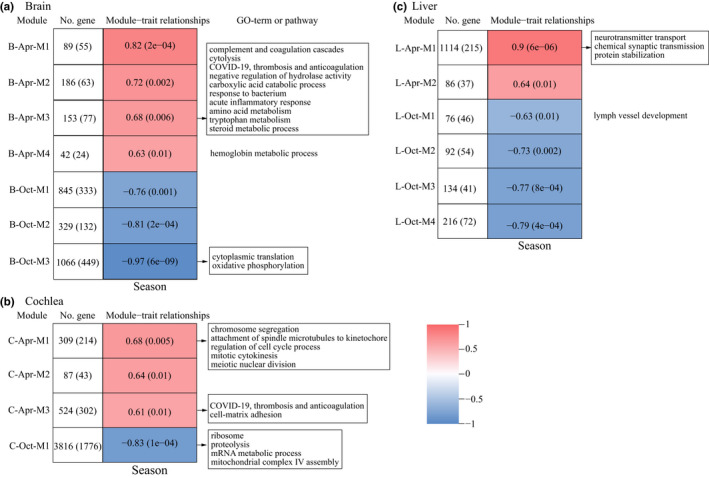
Identification and characterization of modules significantly associated with April and October by WGCNA in the brain (a), cochlea (b), and liver (c). The representative GO terms or pathways identified on hub genes in each module were shown. Each row corresponds to a module with correlation coefficient and significance in parentheses (*p*‐value). Red (positive value) and blue (negative value) colors represent significant associations with April and October, respectively. Number of genes in each module is also listed with the number of hub genes in parentheses

In the brain, for Oct‐specific genes, we found one gene ontology (GO) term in females (GO:0071715, icosanoid transport), whereas for Oct‐biased genes, we identified 35 and 40 GO terms or pathways in females and males, respectively, and most of them were associated with ribosome, cellular respiration, and cholesterol biosynthetic process (Figure 4a and Dryad file Table S4, S5). Weighted gene co‐expression network analysis (WGCNA) identified three modules significantly associated with October (B‐Oct‐M1, ‐M2, ‐M3). However, only hub genes in B‐Oct‐M3 module were enriched into 32 GO terms or pathways and most of them were related to cytoplasmic translation, ribosome, and mitochondrial oxidative phosphorylation (Figure 5a and Dryad file Table S6).

In the cochlea, only one significant GO term was identified in Oct‐specific genes in males (GO:0006910, phagocytosis, recognition), while for Oct‐biased genes, multiple GO terms were found (32 and 26 GO terms or pathways in females and males, respectively) and they were mainly associated with ribosome and electron transport chain in mitochondria (Figure 4b and Dryad file Table S4, S5). WGCNA identified one significant module (C‐Oct‐M1) and its hub genes were enriched into 86 GO terms or pathways mainly associated with ribosome, ATP metabolic process and mRNA metabolic process (Figure 5b and Dryad file Table S6).

In the liver, Oct‐specific genes in males were enriched into two GO terms related to adaptive immune response. For Oct‐biased genes, we found different enrichment results in females and males. Specifically, in females, 156 GO terms or pathways were found and most of them were associated with the immune response, response to reactive oxygen species, insulin signaling, and rhythmic process, whereas in males, 24 GO terms or pathways were identified and most of them were related to ribosomal function and cholesterol biosynthetic process (Figure 4c and Dryad file Table S4, S5). Although WGCNA identified four significant modules (L‐Oct‐M1–M4), hub genes in only one of them (L‐Oct‐M1 module) were enriched into one GO term (GO:0001945, lymph vessel development; Figure 5c and Dryad file Table S6).

Overall, in the brain and cochlea, highly expressed genes in October were mainly involved in cytoplasmic translation in ribosome and cellular respiration via mitochondrial oxidative phosphorylation, whereas in the liver, most genes were associated with the immune response although some of them were also involved in cytoplasmic ribosomal function, insulin signaling, and rhythmic process.

### 3.4 Identification of genes and pathways associated with spring

In the brain, for Apr‐specific genes, we found multiple significant GO terms or pathways associated with the immune response (female: 92; male: 110), such as the complement and coagulation cascades, cytolysis, humoral immune response, and SARS‐CoV‐2 signaling pathway (Figure 4a and Dryad file Table S4), while Apr‐biased genes were enriched into 52 and 10 GO terms or pathways in females and males, respectively, and most of them were involved in the mRNA processing, RNA catabolic process, and protein digestion and absorption (Figure 4a and Dryad file Table S5). WGCNA identified four significant modules associated with April (B‐Apr‐M1–M4) (Figure 5). Functional enrichment analysis on their hub genes revealed one significant GO term in B‐Apr‐M4 module (GO:0020027, hemoglobin metabolic process) and 220 GO terms or pathways in B‐Apr‐M3 module which were mainly related to complement and coagulation cascades, cytolysis, humoral immune response, and metabolism of amino acid, tryptophan, and steroid (Figure 5a and Dryad file Table S6).

In the cochlea, Apr‐specific genes were enriched into multiple GO terms or pathways related to the immune response (female: 10; male: 68) (Figure 4b and Dryad file Table S4), while Apr‐biased genes were mainly involved in the mRNA metabolic process (13 and 73 GO terms or pathways in females and males, respectively) (Figure 4b and Dryad file Table S5). Using WGCNA, we identified three significant modules (C‐Apr‐M1–M3). Hub genes in C‐Apr‐M1 module were enriched into 125 GO terms or pathways and most of them were associated with chromosome segregation and mitotic cell cycle process. In addition, four GO terms or pathways were identified on hub genes in C‐Apr‐M3 module and they were related to COVID‐19, thrombosis and anticoagulation and cell‐matrix adhesion (Figure 5b and Dryad file Table S6).

In the liver, Apr‐specific genes in females were enriched into six GO terms associated with synaptic signaling, while 186 GO terms or pathways were identified in males and most of them were related to muscle system process and regulation of ion transport (Figure 4c and Dryad file Table S4). For Apr‐biased genes, most enriched GO terms or pathways were found to be involved in the electron transport chain in mitochondria and ribosome biogenesis (90 GO terms or pathways in females) (Figure 4c and Dryad file Table S5). WGCNA identified two significant modules (L‐Apr‐M1–M2) and hub genes in one of them (L‐Apr‐M1) exhibited 18 GO terms or pathways mainly associated with neurotransmitter transport and synaptic signaling (Figure 5c and Dryad file Table S6).

Overall, in the brain and cochlea, highly expressed genes in April were mostly associated with the immune responses and metabolic processes, whereas in the liver, most were involved in the cellular respiration, ribosomal function, and synaptic signaling.

## 4 DISCUSSION

To understand the molecular mechanisms of seasonal effects in wild animals, we collected multiple individuals of a bat species (*Rhinolophus sinicus*) sampled in April and October, representing spring and autumn, respectively. To assess tissue‐specific effects, we collected three different tissues (brain, cochlea, and liver) for each individual. Based on a total of 45 RNA‐seq samples, we performed differential expression analysis and Weighted gene co‐expression network analysis (WGCNA) to identify genes and pathways associated with seasonal variations.

### 4.1 Impacts of seasonality on gene expression

We discovered strong seasonal effects on gene expression, in particular in the liver and brain, samples collected in the same season (April or October) tended to cluster together (Figure 2a,b; Figure 3). In addition, we identified a large number of season‐specific and season‐biased genes in all three tissues (Dryad file Figure S1). WGCNA further identified multiple modules significantly associated with each season (Figure 5). Finally, functional enrichment analysis revealed different functional categories for highly expressed genes in each season (Figure 4). A previous study on another hibernating mammal (thirteen‐lined ground squirrel) also revealed seasonal differences of gene expression in the brain between April and October (Schwartz et al., 2013). Comparing to land mammals, strong seasonal effects on gene expression have been widely documented in other groups, such as insects (e.g., *Drosophila*, Zhao et al., 2016; butterflies, Oostra et al., 2018; bees, Bresnahan et al., 2022), birds (Cheviron & Swanson, 2017; Sharma et al., 2020) and aquatic animals (e.g., krills, Höring et al., 2021; dolphins, Van Dolah et al., 2015). Our current work and previous studies in other groups suggest that seasonal gene expression variations might be a general evolutionary response to climate change in wild animals.

In addition, we found that the seasonal effect had a strong sex‐biased pattern with more seasonal genes identified in females than in males (Dryad file Figure S1c) and different functional categories of genes between males and females (e.g., in liver of April, Figure 4c). This might be caused by large physiological differences in females between reproductive and non‐reproductive seasons. However, very few studies have been conducted to explore the sexual difference of gene expression in response to seasonality (but see Macias‐Muñoz et al., 2016). Thus, the generality of this sex‐biased pattern of seasonality observed in this study will require more investigations from other wild animals. Lastly, we found different patterns of seasonal effects across tissues. Compared to the liver, brain and cochlea exhibited similar seasonal effects (see details below).

### 4.2 Characterization of genes and pathways associated with autumn

In this study, we found that highly expressed genes in October (autumn) were mainly involved in cytoplasmic translation via ribosomal function (in all three tissues) and ATP synthesis via mitochondrial oxidative phosphorylation (in brain and cochlea). We speculate that this might be associated with the protein production and accumulation of fat before hibernation in bats. Supporting our speculation, among the Oct‐specific or Oct‐biased genes we found multiple hormone genes which have been proved to play important roles in stimulating food intake and contributing to binge eating behavior (e.g., *RLN3* (relaxin 3), Calvez et al., 2017; *POMC* (proopiomelanocortin), Koch et al., 2015; *NPW* (neuropeptide W), Mondal et al., 2003; *NPY* (neuropeptide Y), Luquet et al., 2005).

In hibernating bats, such as *Scotophilus heathi* and *Myotis ricketti*, oocytes were found to maintain meiotic arrest in autumn (in October and November) and maturation of oocyte usually occurred after hibernation (in April and May) (Chanda et al., 2003; Wang et al., 2008). In this study, we identified a hormone gene (*NPPC*) in the brain with >2‐fold changes of expression in October than in April. Previous studies have shown that NPPC is essential in maintaining meiotic arrest in mouse oocytes (Kiyosu et al., 2012; Zhang et al., 2010). Thus, a higher expression level of this hormone gene in October may help to delay ovulation in female bats.

In the liver, we also found that genes highly expressed in October were enriched into functional categories associated with the immune response (Figure 4c). Among them, *GPX1* (glutathione peroxidase 1), which encodes a cytosolic selenoenzyme (glutathione peroxidase 1), has recently been suggested to play an important role in SARS‐CoV‐2 virulence by interacting with the main protease (M^pro^) of SARS‐CoV‐2 (Seale et al., 2020).

### 4.3 Characterization of genes and pathways associated with spring

Similar to autumn, we also found that multiple highly expressed genes in April (spring) were involved in peptide biosynthetic process via ribosomal function and ATP synthesis via electron transport chain in mitochondria although this was only observed in the liver. This might result from the increase of food consumption after hibernation. Consistent with this proposal, among the Apr‐biased genes in the brain, we also found several hormone genes, which can promote feeding behavior (e.g., *AGRP* (agouti related neuropeptide), Schwartz et al., 2013; *AVP* (arginine vasopressin), Pei et al., 2014).

In addition, the prevalence of cellular respiration in females observed in the liver above might be related to the preparation for the reproduction in April, which may require a large amount of energy. Consistent with this proposal, we found that *DIO2*, the type 2 iodothyronine deiodinase, was highly expressed in April in the liver and brain. This gene plays an essential role in thyroid hormone signaling which can promote seasonal reproduction in birds and mammals (reviewed in Helm et al., 2013; Schwartz & Andrews, 2013).

Similar to autumn, we also found that a large number of genes highly expressed in April were involved in the immune response. However, these immunity genes were mainly identified in the brain and cochlea instead of the liver as in autumn. In particular, some of them were enriched into multiple GO terms or pathways related to COVID‐19. Seasonal patterns of immunity have also been documented in human (Dopico et al., 2015; Goldinger et al., 2015). Consistent with our finding here, a recent study in California has shown that major variations of the immune function in humans correlated with two seasons, late spring and late fall/early winter (Sailani et al., 2020). More importantly, our current study further reveals that seasonal immune response may have tissue specificity.

### 4.4 Clock genes involved in circannual rhythms

It has been proposed that for hibernating mammals, the start and the end of hibernation might rely on expression of clock genes by responding to photoperiod (Helm et al., 2013). Supporting this proposal, in this study, we discovered multiple known core clock genes in both October (before hibernation) and April (after hibernation). Specifically, we first found that multiple female Oct‐biased genes in the liver were enriched into the rhythmic process (GO:0048511) including three of the 16 known core clock genes (*ARNTL* (aryl hydrocarbon receptor nuclear translocator like), *CLOCK* (clock circadian regulator) and *NPAS2* (neuronal PAS domain protein 2), see Wucher et al., 2021 for the whole list of the 16 core clock genes). Among them, *ARNTL* was also found to show a lower expression in April compared to other seasons in the brain of the thirteen‐lined ground squirrel (Schwartz et al., 2013). By searching against the lists of season‐biased genes in October, we also found one additional core clock genes (*RORC*, RAR related orphan receptor C) in liver and two (*CRY2* (cryptochrome circadian regulator 2) and *RORC*) in cochlea.

Similarly, we found multiple core clock genes among highly expressed genes in April, such as *CIART* (Circadian‐associated transcriptional repressor), *NR1D1* (nuclear receptor subfamily 1 group D member 1) and *PER3* (period circadian regulator 3) in liver, *PER3* and *NPAS2* (neuronal PAS domain protein 2) in brain, and *NPAS2* in cochlea. Another important clock gene, *SFPQ* (splicing factor proline and glutamine rich) (see Duong et al., 2011; Johnston et al., 2016), was also observed in all three tissues. It was notable that *NPAS2* and *SFPQ* commonly occurred in all three tissues. Recent studies on circadian process in primates also found that day–night expression variation of *NPAS2* was widespread across 30 tissues in humans (Wucher et al., 2021) and 23 tissues in baboons (Mure et al., 2018). Together with our current results here, we suggest that *NPAS2* might play important roles in both circadian and circannual rhythms. This proposal, however, needs to be tested in more mammal species in the future.

### 4.5 Limitations of the study

First, in this study, we collected seasonal samples from two different years (April 2018 and October 2020). However, gene expression can vary between the same seasons of different years because of changes in phenology caused by rapid global climate changes (Helm et al., 2013). In addition, even if we used seasonal samples in one year, results may still be biased to responses specific to the environmental conditions of the particular year when sampling was conducted. Thus, collecting seasonal samples in different years will be needed in the future to test whether seasonal gene expression variations can be comparable across years. Nevertheless, multiple seasonal genes identified in this study were also documented in other previous studies on mammals (e.g., Mure et al., 2018; Schwartz et al., 2013), including humans (Wucher et al., 2021). Second, the exact ages of individuals used in the current study may be different although we can confidently separate them into adults and nonadults. Because gene expression profiles vary with age (Glass et al., 2013; Somel et al., 2006), individuals with similar ages will be needed to verify seasonal patterns of gene expression observed in this study. This verification can be possible in the future because of a recent promising method to predict the age of bats using DNA methylation profiles (Wilkinson et al., 2021).

## AUTHOR CONTRIBUTION

**Wenli Chen:** Methodology (lead); Project administration (supporting); Writing – original draft (supporting); Writing – review & editing (supporting). **Xiuguang Mao:** Methodology (supporting); Project administration (lead); Writing – original draft (lead); Writing – review & editing (lead).

## CONFLICT OF INTEREST

The authors declare that they have no competing interests.

## Data Availability

All sequencing reads have been deposited in the NCBI Sequence Read Archive (SRA) under Bioproject accession no. PRJNA763734. All supplementary information files are available from the Dryad Repository https://doi.org/10.5061/dryad.gtht76hpp.
